# Differentiation of human iPSCs into functional podocytes

**DOI:** 10.1371/journal.pone.0203869

**Published:** 2018-09-17

**Authors:** Caroline Rauch, Elisabeth Feifel, Georg Kern, Cormac Murphy, Florian Meier, Walther Parson, Mario Beilmann, Paul Jennings, Gerhard Gstraunthaler, Anja Wilmes

**Affiliations:** 1 Division of Physiology, Medical University Innsbruck, Innsbruck Austria; 2 Division of Molecular and Computational Toxicology, Amsterdam Institute for Molecules, Medicines and Systems, Vrije Universiteit Amsterdam, Amsterdam, The Netherlands; 3 Boehringer Ingelheim Pharma GmbH & Co. KG, Nonclinical Drug Safety Germany, Biberach an der Riss, Germany; 4 Institute of Legal Medicine, Medical University Innsbruck, Innsbruck, Austria; University of Houston, UNITED STATES

## Abstract

Podocytes play a critical role in glomerular barrier function, both in health and disease. However, *in vivo* terminally differentiated podocytes are difficult to be maintained in *in vitro* culture. Induced pluripotent stem cells (iPSCs) offer the unique possibility for directed differentiation into mature podocytes. The current differentiation protocol to generate iPSC-derived podocyte-like cells provides a robust and reproducible method to obtain podocyte-like cells after 10 days that can be employed in *in vitro* research and biomedical engineering. Previous published protocols were improved by testing varying differentiation media, growth factors, seeding densities, and time course conditions. Modifications were made to optimize and simplify the one-step differentiation procedure. In contrast to earlier protocols, adherent cells for differentiation were used, the use of fetal bovine serum (FBS) was reduced to a minimum, and thus ß-mercaptoethanol could be omitted. The plating densities of iPSC stocks as well as the seeding densities for differentiation cultures turned out to be a crucial parameter for differentiation results. Conditionally immortalized human podocytes served as reference controls. iPSC-derived podocyte-like cells showed a typical podocyte-specific morphology and distinct expression of podocyte markers synaptopodin, podocin, nephrin and WT-1 after 10 days of differentiation as assessed by immunofluorescence staining or Western blot analysis. qPCR results showed a downregulation of pluripotency markers Oct4 and Sox-2 and a 9-fold upregulation of the podocyte marker synaptopodin during the time course of differentiation. Cultured podocytes exhibited endocytotic uptake of albumin. In toxicological assays, matured podocytes clearly responded to doxorubicin (Adriamycin^™^) with morphological alterations and a reduction in cell viability after 48 h of incubation.

## Introduction

Podocytes, also known as visceral epithelial cells, play a key role in the glomerular filtration barrier and the maintenance of glomerular function [1,2]. Podocyte injury is involved in many human kidney diseases like membranous glomerulopathy and diabetic nephropathy [3]. Due to their relevance in the initiation and progression of nephropathies, podocytes have gained increased attention for their potential role in chronic kidney diseases [4,5].

Podocytes are highly specialized, terminally differentiated cells playing a pivotal role in maintaining the glomerular filtration barrier and producing growth factors for surrounding cells, namely mesangial and endothelial cells [6,7]. They sustain their filtration barrier by wrapping around the glomerular capillaries with interdigitated foot processes, which are connected through intercellular junctions, known as the slit diaphragm. The slit diaphragm facilitates the passing of the primary urinary filtrate and is not traversable for high-molecular-weight plasma proteins [8]. At their apical side podocytes face the primary urine and at their basolateral side they are connected to the glomerular basement membrane via integrins and dystroglycans. The glomerular basement membrane is mainly composed of type IV collagen, laminin, and sulfate proteoglycans which is completed by a fenestrated endothelium [9].

In their mature, fully differentiated state, podocytes have a limited capacity to proliferate *in vivo*, making them vulnerable to various insults leading to glomerular sclerosis [10]. The limited proliferation capacity is further reflected *in vitro*, making it difficult to obtain adequate quantities of podocytes at a differentiated stage for basic research [11–13]. Due to these facts, only a limited number of podocyte cell lines of animal and human origin are available [14,15]. The use of primary podocyte cultures is also limited due to dedifferentiation over time [16]. However, there is a clear need for meaningful podocyte culture systems [17–19].

A conditionally immortalized human podocyte cell line has been established by transfection of podocyte primary cells with a temperature-sensitive SV40 large-T antigen gene construct [15]. However, differentiation of cells at the non-permissive temperature of 37 °C bears phenotypic variations within different podocyte cell isolates [20]. The development of new technologies to reprogram somatic cells into the pluripotent state, so-called induced pluripotent stem cells (iPSCs) [21], opened new avenues to re-differentiate iPSC into tissue cells of all 3 germ layers [22]. Thus, iPSCs generated from adult somatic cells provide an almost unlimited supply of human cells without ethical concerns known for embryonic stem cells. iPSCs could be used in biochemical research for disease modelling, in the development and screening of drugs, and in toxicity testing, which may lead to promote personalized medicine and future applications in regenerative medicine for cell replacement therapy [23–29]. Furthermore, iPSCs offer exciting opportunities to create kidney organoids that enable *in vitro* podocyte research [30–33].

In this respect, the directed differentiation of human iPSCs into glomerular podocytes was described in two recent studies [34,35]. Song et al. [35] used a 10 day directed differentiation with an intermediate suspension culture of mechanically dissociated cells, while Ciampi et al.[34] applied a three-stage protocol including induction into intermediate mesoderm, commitment towards nephron precursors, and specification into podocytes. iPSC-derived podocytes were characterized by the expression of podocyte-specific markers, the endocytic internalization of albumin, and the disappearance of pluripotent markers Oct3/4 and Sox-2.

The aim of the current study was to evaluate the reproducibility and robustness of currently available podocyte differentiation protocols and to optimize the protocols accordingly. Here, we report on a direct differentiation of human iPSCs into functional podocytes, based on the protocols of Ciampi et al. [34] and Song et al. [35]. A modified, robust and reproducible differentiation protocol is described, tailored to different human iPSC lines, generated from adult and neonatal donors, that were reprogrammed by Sendai virus [36,37] during the course of the IMI-funded StemBANCC project (http://stembancc.org) [23]. iPSC-derived podocytes exhibited distinct morphological features of podocyte foot processes, and expressed the podocyte-specific markers synaptopodin and nephrin. Beyond recent studies[34,35] differentiated podocytes presented here expressed clear functional features by their sensitivity to doxorubicin emphasizing their mature-like state of differentiation.

## Results

The differentiation protocol developed in the present study is based on a direct path to mature podocyte-like cells without intermediate progenitor stages, using a combination of BMP7, activin A and retinoic acid. In modification of earlier protocols [34,35], the amount of fetal bovine serum (FBS) in culture media was reduced to a minimum, and thus β-mercaptoethanol (β-ME) could be omitted. In studies on chemically defined culture conditions for human iPSCs it was shown, that β-ME is toxic when high molecular weight protein components, like albumin (BSA), are removed from the culture medium [38]. Without β-ME, BSA was no longer necessary for human iPSC culture, suggesting that β-ME is inactivated by unspecific protein binding. Therefore, by decreasing the content of FBS down to 1.25% (v/v) in the present culture media, β-ME was no longer added. Further modifications that were made to the protocol include the omittance of the suspension cultures on days 1–3. In the current protocol the transfer of cells from ultra-low cluster plates to gelatin-coated dishes was no longer needed. This enabled a more standardized procedure since the seeding of a precise number of cells was now possible. The seeding density was actually one of the main factors contributing to the reproducibility of the present protocol.

### The initial seeding density is crucial for differentiation efficiency

**Fig 1:**
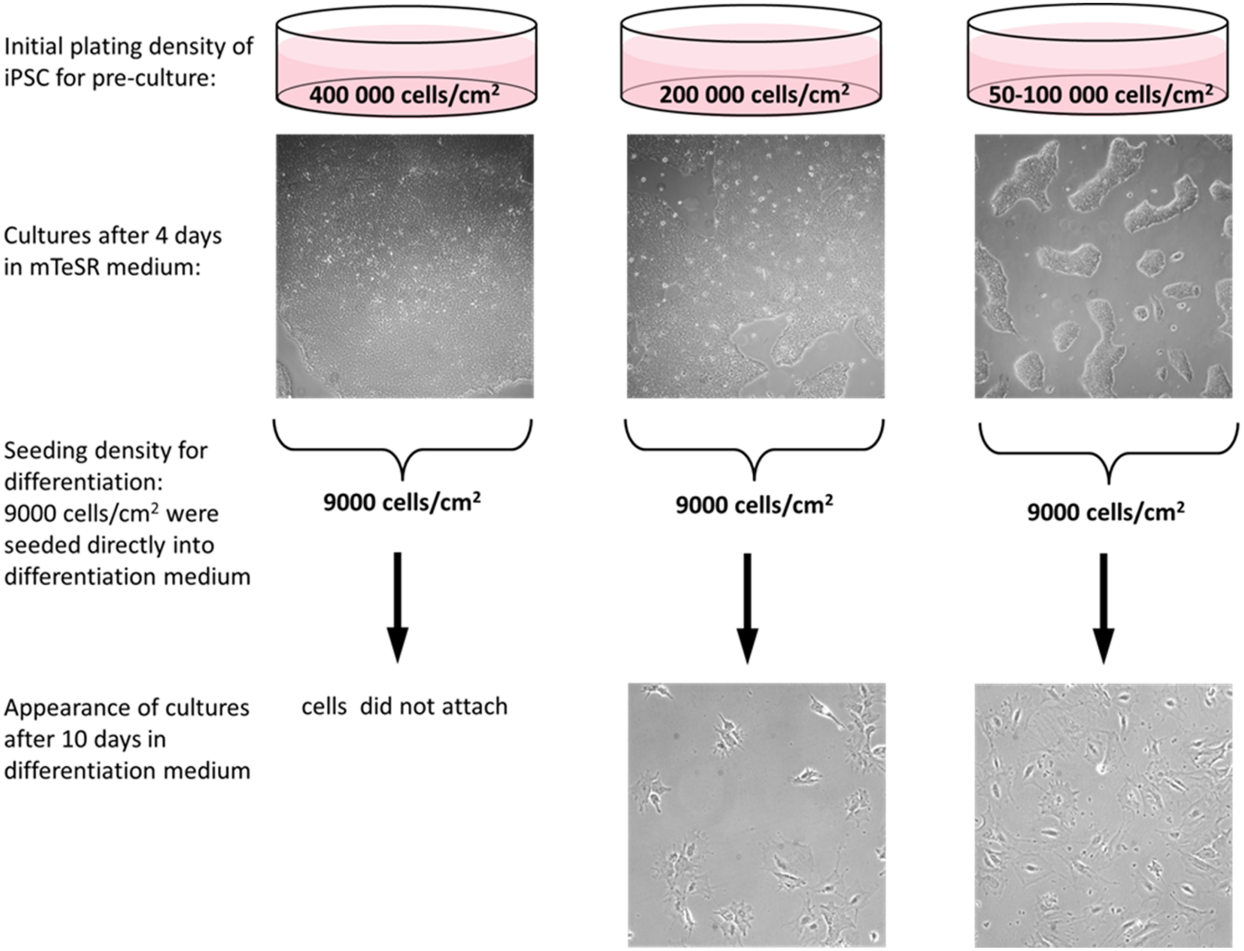
Effect of initial cell plating density of undifferentiated iPSC for podocyte differentiation efficiency. Initial cell density of approximately 100 000 cells/cm^2^ (right column) is optimal for podocyte differentiation. For differentiation 9000 cells/cm^2^ were directly seeded into differentiation medium. Representative images of the SBAD3 iPSC line are shown.

In systematically elaborated culture experiments it was found, that the plating densities of pre-cultures of undifferentiated iPSCs as well as the seeding density of the differentiation cultures are absolutely crucial for a stable and reproducible differentiation of iPSCs into podocyte-like cells. Several initial iPSC plating densities of undifferentiated cells as well as several cell seeding densities for differentiation cultures were tested. Initial plating of undifferentiated iPSC density was tested at 50–100,000, 200,000 and 400,000 cells/cm^2^. After 4 days in culture, iPSCs were detached by Accutase to yield single cell suspensions to start differentiation into podocyte-like cells. Cells were counted and 9,000, 15,000 and 40,000 cells/cm^2^ were then plated on Geltrex-coated multi-well plates for differentiation experiments. The results of the initial plating experiments are shown in Fig 1. The most efficient seeding densities for generating iPSC-derived podocytes were 50–100,000 cells/cm^2^ for the undifferentiated iPSC pre-cultures, and after 4 days in culture, 9000 cells/cm^2^ for efficient differentiation into podocyte-like cells, respectively. The elaborated plating protocol ensured optimal growth conditions for a maximum of evenly distributed cells at appropriate density for cell enlargement and development of podocyte-like structures, respectively. It also ensured sufficient cell numbers and biomass to be obtained for down-stream experiments. These seeding densities were further critical for the reproducibility and robustness of the protocol, as higher seeding densities of undifferentiated iPSCs at 200.000 and 400.000 cells/cm^2^ resulted in significantly lower numbers of podocyte-like cells or no differentiation into podocyte-like cells, since cells did not attach on day 1 (Fig 1). This protocol was successfully repeated with three additional iPSC lines (SBAD2, SBNEO and SFC018; see below).

### Differentiation of human iPSC into podocyte-like cells

Four different iPSC lines were used for differentiation: SBAD3-01(SBAD3); SBNEO3-01 (SBNEO); SFC018-03-0(SFC018) and SBAD2-01 (SBAD2). After detachment with Accutase, single iPS cells were gently suspended directly in differentiation medium (medium M1), containing activin A, bone morphogenetic protein (BMP7), and retinoic acid and seeded on growth factor reduced basement membrane matrix (Geltrex)-coated surface. After ten days in differentiation media, differentiated podocyte-like cells could be further maintained for at least another 10 days in maintenance medium (medium M2), which was devoid of differentiation factors activin A, BMP7, and retinoic acid (DMEM/Ham F-12 with 2.5% FBS, 100 μM NEAA and penicillin/streptomycin) for further research.

**Fig 2:**
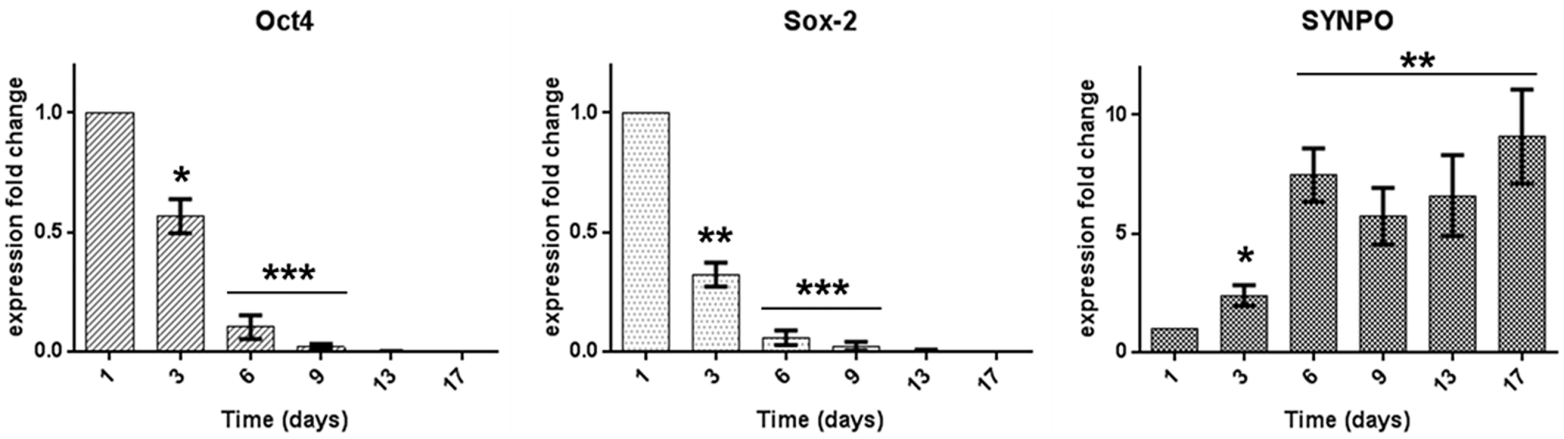
Time course of specific gene expression during differentiation of iPSC into podocyte-like cells, assessed by qPCR. The pluripotency markers Oct4 and Sox-2 gradually decreased to zero at day 13, while podocyte marker synaptopodin (SYNPO) mRNA specifically increased 9-fold during the course of differentiation. Data are given as means ± SEM of 7 independent series of experiments, pooled from the 3 iPSC lines (SBAD3, SBNEO, SFC018) (*P < 0.05; ** P < 0.01; *** P < 0.001).

The time course of differentiation of iPSCs into podocyte-like cells was monitored by determination of mRNA expression profiles of the pluripotency markers Oct4 and Sox-2, and of synaptopodin, a podocyte-specific marker, by quantitative real-time PCR (qPCR). As depicted in Fig 2, characteristic time courses of the specific markers were found. While Oct4 and Sox-2 gradually decreased to zero during the first 9 days of differentiation, synaptopodin mRNA expression levels gradually increased 9-fold after 17 days of cells in medium M2.

### Morphology of iPSC-derived podocyte-like cells

**Fig 3:**
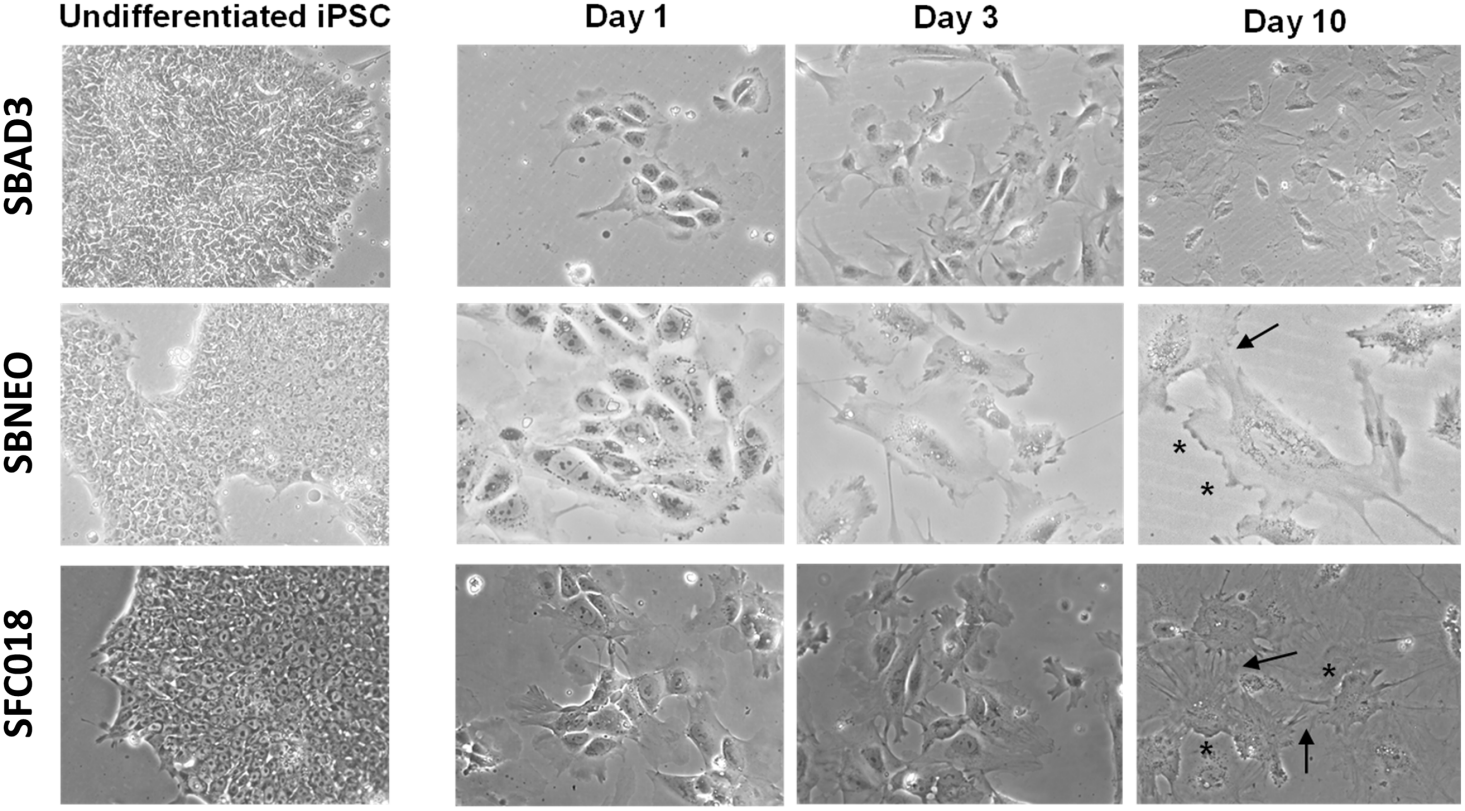
Morphological changes of cells during differentiation of human iPSC into podocyte-like cells. Phase contrast images of iPSC during differentiation. Podocyte-like foot processes are indicated with an asterisks and interdigitations between foot process with an arrow.

**Fig 4:**
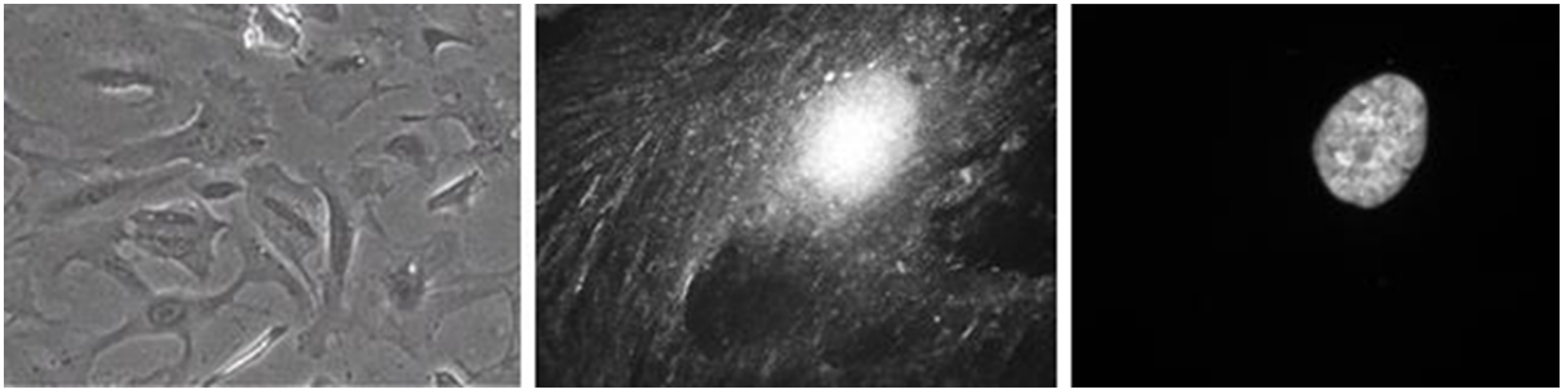
Differentiated conditionally immortalized human podocytes served as reference controls. The podocyte cell line (Saleem et al., 2002) was cultured under non-permissive conditions. Phase contrast images of cell morphology (left) and synaptopodin immunofluorescence staining (middle). Right: Staining of cell nucleus with Hoechst 33342.

The temporal changes of iPSC during differentiation towards a podocyte-like morphology were assessed by phase-contrast microscopy (Fig 3). Cells changed from a typical iPSC colony morphology, consisting of small rounded cells, to enlarged cells with the characteristic foot processes. After ten days of differentiation, cells clearly showed long spindle like projections, short rounded projections, fine processes, and interdigitations between cells. Differentiated podocyte-like cells could be maintained in culture for at least further 10 days. Conditionally immortalized human podocytes [15] served as reference controls. When these cells were expanded at the permissive temperature of 33 °C, they remained in undifferentiated, epithelial-like state (not shown); however, when transferred to 37 °C, they started to differentiate into distinct podocyte morphology with well-preserved foot processes and synaptopodin expression (Fig 4). Thus, both iPSC derived podocyte-like cells and conditionally immortalized human podocytes showed comparable podocyte-specific morphologies after terminal differentiation.

### Expression of podocyte-specific markers

**Fig 5:**
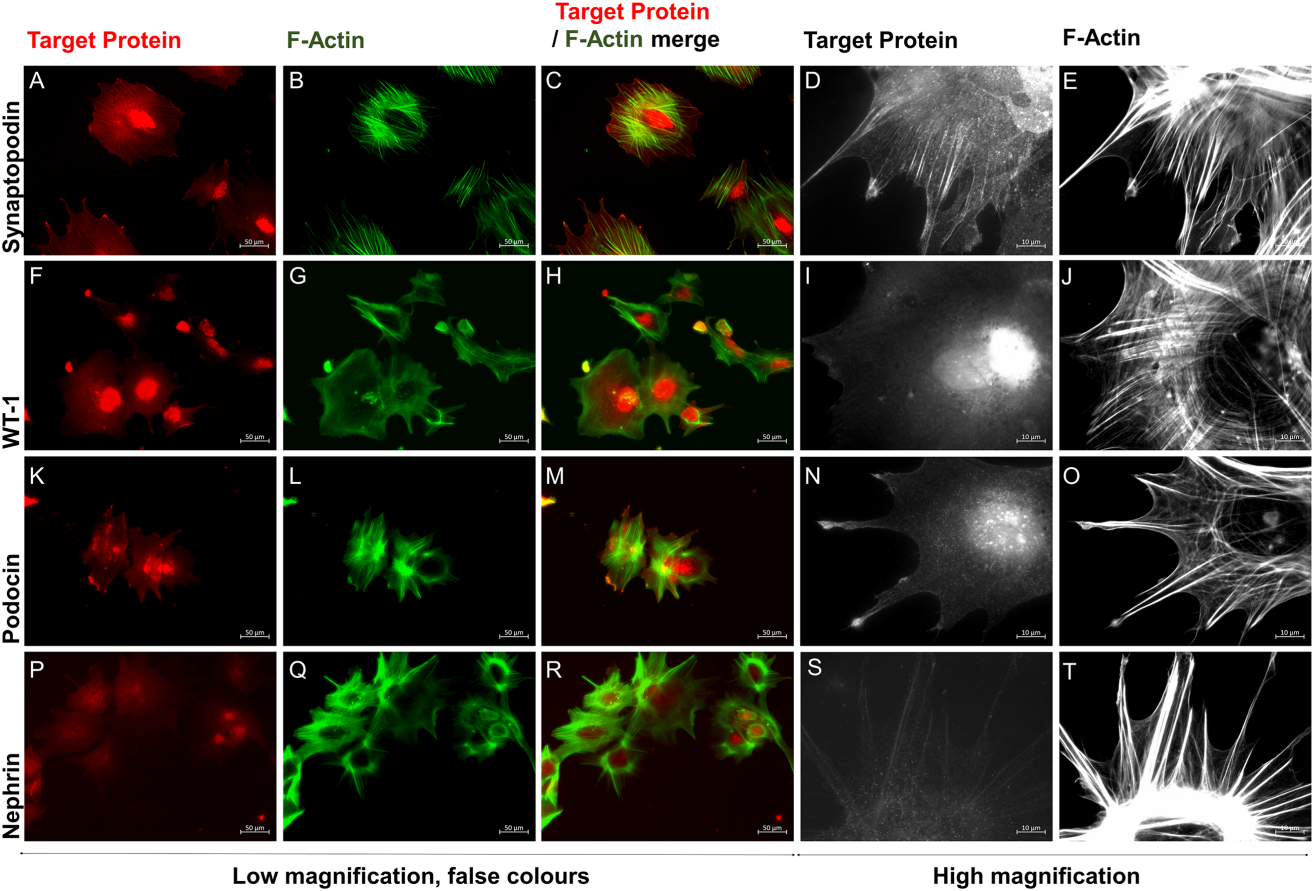
Immunostaining of podocyte-like cells derived from the iPSC line SBAD3 with podocyte markers and F-actin staining. iPSC were differentiated on glass cover slips fixed and stained for synaptopodin, WT-1, podocin, and F-actin as described in methods. Red and green colours were applied post capture. Staining in two other iPSC lines are provided in S1 and S2 Figs.

Aside from the distinct podocyte-specific morphology, the protein expression of podocyte-specific markers by differentiated iPSC-derived podocyte-like cells and human immortalized podocytes, respectively, was determined by *immunofluorescence* and Western blot anaylsis. Synaptopodin, an actin-associated protein (see also Fig 2), podocin and nephrin, both raft-associated components of the glomerular slit diaphragm, and the transcription factor WT-1 could be detected by immunofluorescency microscopy (Fig 5, S1 and S2 Figs). While cellular location of synaptopodin, podocin and WT1 were as expected, nephrin staining appeared in the cytoplasm and was not localized within the slit diaphragm of the podocytes. Since synaptopodin binds specifically to actin in the foot processes of glomerular podocytes [14], F-actin cytoskeletal bundles were visualized in differentiated podocyte-like cells with fluorescent-labeled phalloidin [14,39]. Representative micrographs are shown in Fig 5 and S1 and S2 Figs.

**Fig 6:**
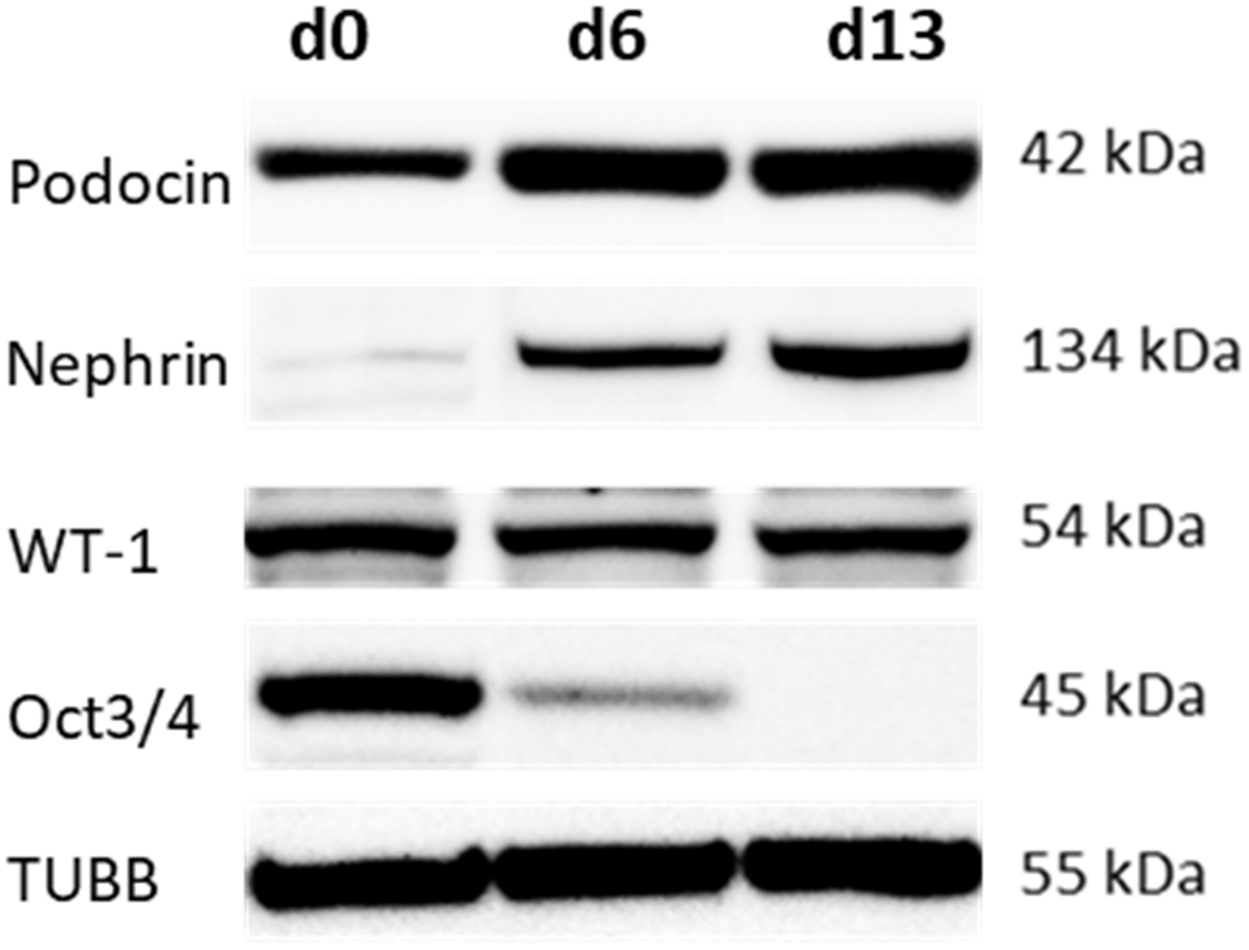
Time course of induction of podocyte-specific markers during differentiation. Differentiating, SBAD3 iPSC were lysed at days 0 (undifferentiated iPSC, d0) and at days 6 and 13 and processed for Western blot analysis. Representative blots are shown. Expression levels for two other iPS cell lines are shown S3 Fig.

Furthermore, the induction of podocyte-specific marker expression during the course of differentiation was determined by Western blot analysis. Time course experiments with three iPSC lines were performed in triplicate. Representative blots are shown in Fig 6 and S3 Fig. The iPSC-derived podocyte-like cells exhibited increasing protein expression of the podocyte-specific markers podocin and nephrin during the time course of differentiation up to day 13. For WT1, which is also expressed in undifferentiated iPSC, expression levels were maintained during the differentiation time course. Oct3/4 protein expression declined to undetectable levels at day 6. This was comparable with mRNA levels as measure via PCR (Fig 2).

In order to test for podocyte-specific functions of differentiated cells, two functional assay regimes were applied: (a) the endocytic uptake of albumin and (b) the susceptibility of podocyte-like cells to doxorubicin (Adriamycin^™^).

### Endocytosis of albumin by podocyte-like cells

**Fig 7:**
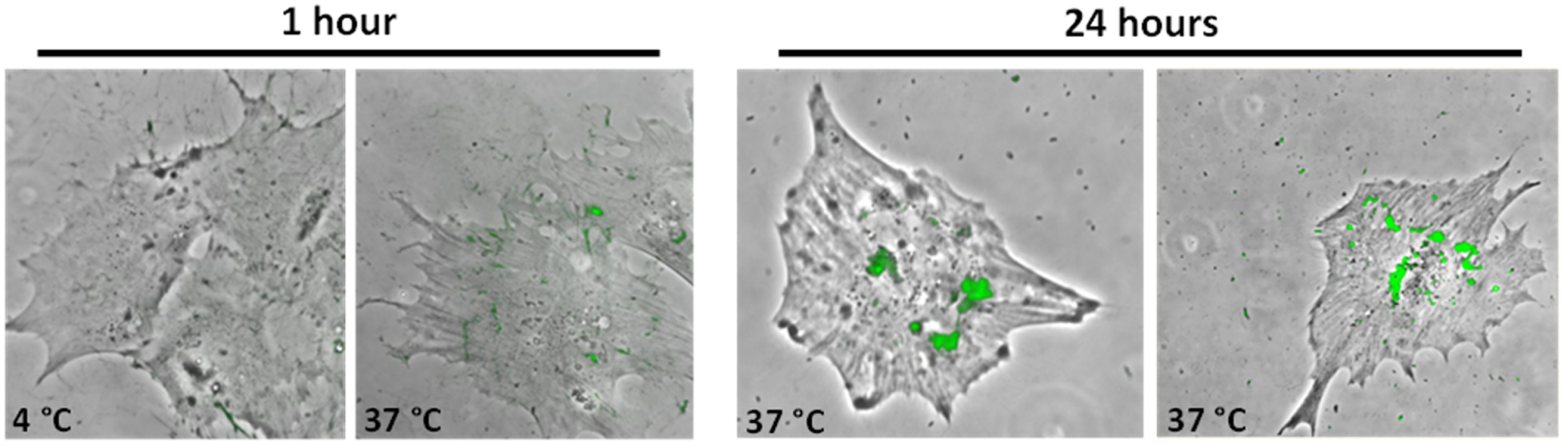
Albumin endocytosis by SBAD3 iPSC-derived podocyte-like cells on day 10 of differentiation. Cells were grown and differentiated in 12-well plates and incubated with FITC-albumin for 1 h at 4 °C and 37 °C and for 24 h at 37 °C. Representative images of two independent experiments are shown.

The ability of endocytic uptake of FITC-labeled albumin was investigated by fluorescence microscopy. Serum-starved, fully differentiated podocyte-like cells were incubated with 1 mg/ml FITC-conjugated bovine serum albumin for 1 h at 4°C and 37°C, respectively, and for 24 h at 37°C. As shown in Fig 7, fluorescence microscope images show a clear temperature-dependent endocytosis of albumin after 1 h and 24 h of incubation at 37 °C. No endocytic uptake of labeled albumin was observed at 4°C for 1 h.

### Podocytes are a specific target for doxorubicin (Adriamycin)

**Fig 8:**
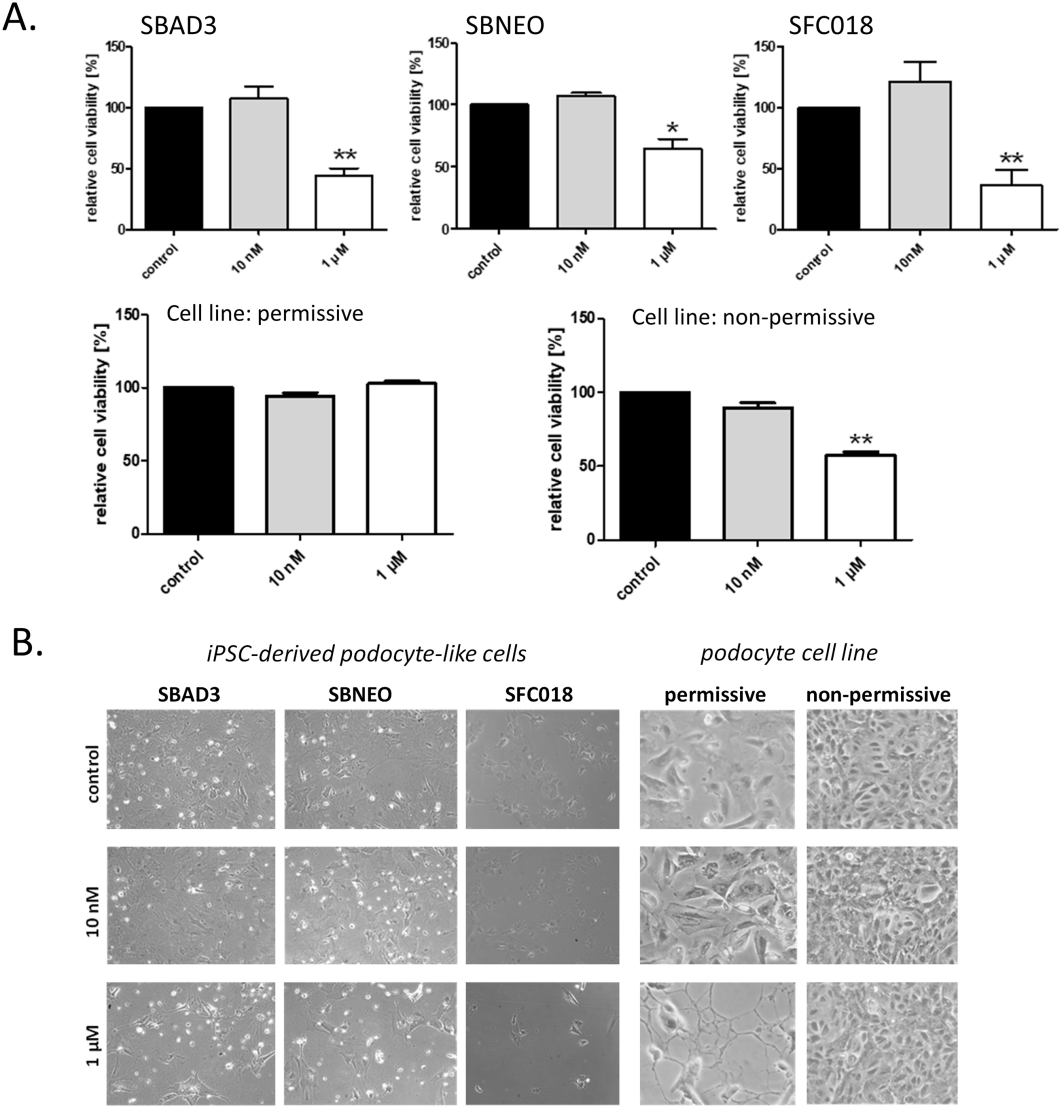
Effect of doxorubicin exposure on the viability and morphology of iPSC derived podocytes compared to a podocyte cell line. SBAD3, SBNEO and SFC018 iPSC-derived podocyte-like cells and human conditionally immortalized podocytes were incubated for 48 h in 6-well plates. Cell viability was assessed by resazurin assay (**A**). Results are expressed as means ± SEM, *P<0.05; **P<0.01. Phase contrast micrographs (**B**). Original magnification: iPSC-derived podocytes 50 X and the cell line 10 X.

Doxorubicin (DOX), a nephrotoxic anti-cancer agent is, aside its cardiotoxicity, known to be specifically toxic for glomerular podocytes. DOX leads to podocyte foot process effacement and increased glomerular permeability causing proteinuria. DOX-associated nephropathy is caused by interfering with actin reorganization of podocytes [40] and mitochondrial dysfunction [41]. Therefore, the impact of DOX on differentiated podocyte-like cells was used as another functional endpoint. Cultures of iPSC-derived differentiated podocyte-like cells and of undifferentiated and differentiated human immortalized podocytes were incubated with DOX for 48 h at a concentration of 10 nM and 1 μM (Fig 8). While 10 nM DOX showed no effect on either cell type, 1 μM DOX caused a drastic decrease in cell proliferation rates in differentiated podocyte-like cells, whereas undifferentiated human podocytes were unaffected, indicative for the susceptibility of differentiated, mature podocytes to DOX intoxication.

## Discussion

Podocytes are key cells in the formation of the glomerular filtration barrier. In the complex filtration barrier network of the glomerulus, podocytes are the most vulnerable components. Most glomerulopathies are associated with podocyte injury causing various types of diseases, that may account for approx. 90% of end-stage kidney disease [42]. When podocytes are injured, their main function for sieving macromolecules cannot be longer maintained. Disruption of the complex podocyte structure or reduction in the number of podocytes results in proteinuria [43]. Podocyte disruption or loss are involved in renal diseases like focal segmental glomerulosclerosis (FSGS) or diabetic nephropathy [5,6].

Thus, the central role of podocytes in glomerular dysfunctions necessitates reliable model systems to study normal and impaired podocyte functions at the cellular and subcellular level. In this respect, *in vitro* cultures of human podocytes gained specific importance. However, there are intrinsic difficulties of growing differentiated podocytes in *in vitro* culture, that have been discussed extensively [11–13]. Mature podocytes are terminally differentiated cells. This is a profound limitation in cell culture, since growth arrested, differentiated cells do not proliferate and obtaining cells in sufficient quantities for basic research is extremely difficult. To circumvent this problem, undifferentiated podocyte “precursor” cultures should be expanded to a certain cell density, and then be differentiated by specific stimuli. Two strategies were pursued: 1. conditionally immortalized podocytes of human origin and 2. induced pluripotent human stem cells (iPSCs).

The first strategy was applied using a conditionally-immortalized human podocyte cell line carrying a temperature-sensitive mutant of the SV40 large-T antigen [15]. Thereby the cells can be expanded at the permissive temperature of 33 °C to an appropriate cell density. Thereafter, when switched to the non-permissive temperature of 37 °C, the cells enter growth arrest and start to differentiate into mature podocytes, exhibiting foot processes and expressing synaptopodin, nephrin and podocin (Fig 4). However, although conditionally immortalized, these cells were only usable for approx. 20 passages, when new human glomerular primary cultures had to be initiated, expanded and transfected/immortalized for further propagation [13,15].

The second strategy is the employment of human iPSC. In 2007 Takahashi and Yamanaka discovered a technique to reprogram adult human cells into pluripotent stem cells [21], which enables a non-controversial, ethical acceptable method to differentiate pluripotent cells into any human somatic cell type. iPSCs are, by definition, immortal and can be expanded indefinitely. This paved new ways for investigating diseases (“disease in a dish”) [23,24,27,28,44], for *in vitro* toxicity testing and drug safety assessment, and for tissue engineering and cell therapy applications [25,29,45].

Furthermore, human iPSC opened the possibility of directed differentiation into all cell types of the nephron [30]. Recent strategies follow the *in vivo* stages of embryonic kidney development, with differentiation of iPSC into intermediate mesoderm and metanephric mesenchyme, and the induction of nephron progenitor cells, thereby recapitulating embryonal developmental stages of the kidney *in vitro* [31,46,47].

Here we present a method to directly differentiate human podocytes from human induced pluripotent stem cells (iPSCs) in a one-step protocol, using a cocktail of retinoic acid, activin A, and BMP7. Already published protocols [34,35] were modified for adherent cell culture systems. As mentioned above, for the present protocol the culture medium content of FBS was lowered to a minimum of 1.25% (v/v) in differentiation medium M1 and 2,5% (v/v) in maintenance medium M2, respectively, and therefore β-ME could be omitted.

Characteristic podocyte morphology, such as long spindle-like projections, fine processes, short rounded projections, and interdigitations between cells could be observed on day 10 of differentiation or earlier (Fig 3). The immunofluorescence expression of podocyte markers synaptopodin, an actin-associated protein, podocin and nephrin, components of the slit diaphragm, and of the transcription factor WT1 could be shown (Fig 5, S1 and S2 Figs). Podocin, nephrin and WT1 could be further verified by Western blot analysis (Fig 6 and S3 Fig) and synaptopodin by qPCR (Fig 2) at different time points.

qPCR results showed a gradual decrease in mRNA levels of pluripotency markers Oct4 and Sox-2, paralleled by an increase in podocyte marker synaptopodin mRNA during iPSC differentiation (Fig 2). Oct4 and Sox-2 are specific markers for the pluripotent state of embryonic stem cells. The essential role of Oct3/4 and Sox-2 in the maintenance of the pluripotent state is further substantiated by the crucial role of the transcription factors in the reprogramming of somatic cells into iPSCs [21]. Podocin and nephrin also showed a differentiation induced increase in expression at a protein level. WT1 however, is also expressed in undifferentiated iPSC and its expression was maintained during differentiation.

Variations between iPSC lines are well documented. Regardless of the source of somatic cells or the method chosen, the reprogramming process itself is influenced by a number of variables that affect the reproducibility and quality of the resulting iPSCs. Each iPSC line can be considered to be a distinct clone [48]. The reproducibility and robustness of the present protocol was proven on four different iPSC lines developed during the course of the IMI-funded StemBANCC project (http://stembancc.org) [23]. iPSCs from reprogrammed fibroblasts of adult (SBAD2 and SBAD3) and neonatal origin (SBNEO), as well as of a migraine patient (SFC018), respectively, were differentiated resulting in well comparable podocyte-like cells, in terms of morphology (Fig 3) and expression of podocyte-specific markers (Figs 5 and 6). Since iPSCs can be obtained from any individual [29], the generation of iPSC from patients with e.g. genetic diseases [45] offers potential opportunities for human disease modelling. Patient-specific iPSCs enable in the future to study the pathophysiology of the disease as well as the response to drugs *in vitro* at the cell level [24,27].

Another major finding of the present study was that the plating density of the undifferentiated iPSC stocks, as well as the seeding density for differentiation, respectively, are absolutely crucial for a sufficient and proper differentiation efficiency. Elaborated plating protocols were mandatory for differentiating evenly distributed iPSC into consistently spread podocytes at well-defined cell densities. An initial plating density of 50–100,000 cells/cm^2^ was optimal for pre-cultures of undifferentiated iPSC. High cell numbers in dense cultures of iPSC result in rapid acidification of the mTeSR1 medium. We have recently shown that high cell densities can decrease the culture medium pH to 6.8 within 24 h. At this low pH, undifferentiated iPSC enter a transient growth arrest in G0/G1-phase [49]. This is likely to have an impact on subsequent differentiation and should therefore be avoided with low initial seeding densities. A subsequent seeding of 9,000 cell/cm^2^ were proven to be most efficient for terminal differentiation (Fig 1). Cells need space for proper differentiation. When iPSC differentiate into podocyte-like cells, they substantially increase in size (Fig 3) and exhibit a more complex morphology with slit diaphragm-like cell contacts (Fig 4). Overcrowding of undifferentiated cells is one of the major pitfalls in podocyte culture [11]. For growth-restrictive conditions of immortalized podocytes, the authors also recommend plating densities of 5,000 to 10,000 cells/cm^2^.

The differentiation protocol applied in the present study involved an inducer cocktail, containing retinoic acid, BMP7, and activin A. Retinoic acid is known to have an anti-proliferative and pro-differentiation effect in many tissues, including the kidney and especially the podocyte [31,50], and has a clear impact on the podocyte phenotype *in vitro* and *in vivo* [51]. It is crucial for differentiation towards intermediate mesoderm and regulates the expression of the transcription factor WT-1, which is required for the expression of several podocyte genes [52]. WT-1 is only expressed in podocytes of the mature kidney [7,53]. BMP7 acts on podocytes in an autocrine, paracrine and endocrine manner, it phosphorylates Smad5, which is involved in podocyte differentiation and survival. It is expressed in many tissues during development including podocyte precursor cells [54]. BMP7 is a potent inducer of OSR1 expression, a specific intermediate mesoderm marker [55]. Activin A is needed for induction of intermediate mesoderm [56].

The ability of endocytotic uptake of albumin is a well described feature of differentiated podocytes [57,58]. Albumin endocytosis by differentiated podocytes was investigated by incubation of cells with FITC-labelled albumin for 24h at 37 °C and for 1h at 37 °C (uptake) and 4 °C (no uptake). After 1h and 24h of incubation at 37 °C the specific, temperature-dependent endocytic uptake of albumin could be detected via fluorescence (Fig 7).

In functional assays, doxorubicin, an anti-cancer chemotherapeutic drug, showed a toxic effect on differentiated iPSC-derived podocytes. Doxorubicin at 1 μM resulted in a 50% reduction in cell viability of iPSC-derived podocyte-like cells after 48h of exposure. This was comparable to immortalized podocytes cultured under non-permissive, but not permissive conditions (Fig 8). Recently, doxorubicin-induced podocyte injury was also demonstrated in a glomerulus-on-a-chip model using iPSC-derived podocytes [59].

To summarize, the present investigation demonstrates a direct method to generate podocyte-like cells from human iPSCs. This represents a minimal invasive and ethically accepted way to obtain human podocytes in appropriate quantities for basic research and offers new possibilities for *in vitro* toxicity studies and drug safety assessment. Control and patient iPSC-derived mature human podocytes in monolayer culture may also facilitate new avenues for drug development and development of personalized therapeutic strategies.

## Material and methods

### Cell culture

Human iPSCs used in the present study were obtained during the course of the IMI-funded StemBANCC project (http://stembancc.org) [23]. Human fibroblasts of adult (SBAD3 clone 1 and SBAD2 clone 1) and neonatal (SBNEO clone 1) origin (Lonza) and primary fibroblasts of a migraine patient (SFC018 clone 03–01) were reprogrammed by non-integrating Sendai virus transfection [36,37,60] according to project-specific SOPs. Reprogramming efficiency and cell status was periodically verified by the iPSC producing laboratory. Culture and propagation of iPSC adhered to specific SOPs that have been elaborated within the StemBANCC consortium. iPSC were routinely cultured on matrigel-coated plates (Geltrex, GIBCO) in mTeSR^™^1 medium (StemCell Technologies). Cultures were passaged twice a week with Versene (Lonza) at a split ratio of 1:4 to 1:12.

Periodical cell authentication of the four iPSC lines used in the present study was performed by short tandem repeat (STR) profiling [61] as described recently [49]. In brief, cellular DNA was isolated (Qiagen Puregene Cell Kit, Cat.No. 158745) and STR profiles were analyzed with the Promega PowerPlex 16 HS System in an Applied Biosystems Genetic Analyzer and GeneMapper ID-X 1.2 software.

Conditionally immortalized human podocyte-like cells, generously provided by Prof. M. Saleem, Bristol, UK [15] served as reference controls. Cells were cultured on collagen type I-coated dishes (BioCoat^™^, BD Biosciences) at the permissive temperature of 33 °C in RPMI 1640 medium, supplemented with 100 U/ml penicillin, 0.1 mg/ml streptomycin, glutamine, and insulin, transferrin, sodium selenite mixture (ITS) (Sigma-Aldrich), and 10% fetal bovine serum (GIBCO) as recently described [62]. At 70–80% confluence, cultures were switched to 37 °C to induce growth arrest and subsequent differentiation (non-permissive condition), respectively, and were further maintained for 2 to 3 weeks.

### Differentiation of iPSC into functional podocytes

#### The critical role of initial cell density

During a series of experiments it was observed, that the initial cell densities of stock iPSCs as well as the seeding densities of the differentiation cultures play a critical role in order to obtain reproducible results in the differentiation of iPSC into podocytes. To this end, pre-cultures of undifferentiated iPSC were plated at densities of 50–100,000, 200,000 or 400,000 cells/cm^2^. After 4 days, cultures were passaged with Accutase (GIBCO) to obtain single cell suspensions, and iPSCs were seeded on plates coated with Geltrex at a density of 9,000, 15,000 and 40,000 cells/cm^2^ for differentiation. In order to avoid uneven concentric distribution of cells in multiwell plates, and thus non-optimal conditions for a proper development of foot processes and interdigitations between adjacent/neighbouring cells, cells were seeded with half of the recommended volume of media per well. Back and forward and left and right gentle shaking movements were performed instead of gentle rotation before placing the plates into the incubator. The residual media was added after 5h, when cells were attached.

#### Differentiation protocol

In the present one-step protocol undifferentiated iPSCs were seeded at the cell densities described above into differentiation medium containing 5 μM Rho-associated kinase (ROCK) inhibitor Y-27632 (Abcam, Cat. No. ab120129) for 24 hours. Differentiation medium (medium M1) consisted of DMEM/Ham F-12 (GIBCO Cat. No. 31330–038), supplemented with 1.25% FBS (GIBCO Cat. No. 10270), 100 μM nonessential amino acids (NEAA), 15 ng/ml BMP7, 10 ng/ml activin A, and 100 nM retinoic acid (all from ThermoFisher Scientific), and 100 U/ml penicillin, 0.1 mg/ml streptomycin (Sigma). After 24 h, ROCK inhibitor was removed by feeding the cells with medium M1 and cultures were further maintained in M1 and fed every 2–3 days. After ten days, differentiated cells were further incubated for another 10 days in maintenance medium (medium M2), without presence of differentiation growth factors (DMEM/Ham F-12 with 2.5% FBS (GIBCO), 100 μM NEAA and 100 U/ml penicillin, 0.1 mg/ml streptomycin). Media were changed every 48 hours.

### Quantitative real-time PCR (qPCR)

Total RNA was isolated with QIAzol (QIAGEN, Cat. No. 79306) from undifferentiated iPSC cultures, and from podocyte-like cell cultures at day 1, 3, 6, 9, 13 and 17 in differentiation media. 50 ng of total RNA were used for first-strand cDNA synthesis with Superscript II (Life Technologies), following the manufacturer’s instructions.

Quantitative real time PCR (qPCR) was performed using TaqMan Gene Expression Master Mix (Applied Biosystems). Assays for synaptopodin (SYNPO, Hs00702468_s1), Oct4 (Hs04260367_gH) and Sox-2 (Hs01053049_s1) were purchased (TaqMan Gene Expression Assays, Life Technologies), based on the supplier’s validated bioinformatics pipeline for optimal alignment to the selected genes. Threshold cycle values and expression levels, respectively, were normalized against endogenous 18S RNA (Applied-Biosystems VIC^™^/TAMRA^™^ probe, cat. 4310893E, Life Technologies).

### Immunocytochemistry

Cells were differentiated on glass coverslips for 10 days and fixed with 4% para-formaldehyde in PBS for 10 min at room temperature (RT). After washing three times with PBS, cells were incubated for 30 minutes in blocking buffer (PBS with 5% BSA, 1% Triton X-100). Cultures were stained with primary antibodies, anti-synaptopodin (Abcam ab224491) at a dilution of 1:75, anti-NPHS2 (podocin, Sigma P0372) at 1:500, anti-NPHS1 (nephrin, Abcam ab183099) at 1:75 and WT-1 (R&D Bio-techne, AF5729) at 1:100, and incubated at RT for 30 min. The secondary antibody Alexa Fluor 568 (Thermofisher; Goat: A-11056, Rabbit: A-10040) was incubated at a dilution of 1:250 for 1 h. Either F-actin or the nucleus was counter stained using phalloidin (ActinGreen^™^ 488, GeneCopoeia, Rockville, MD) or Hoechst 33342 respectively. Fluorescent images were processed using ImageJ.

### Western blot analysis

Cells were harvested at different time points and homogenized in RIPA buffer (Sigma) containing a protease inhibitor cocktail (Sigma). Protein concentrations were determined using a BCA assay (PIERCE). Equal amounts of protein (15–50 μg/well) were subjected to SDS-PAGE (4–7% NuPAGE Bis-Tris gels, Invitrogen), and transferred to PVDF membranes. After blocking in TBST with 5% non-fat milk, membranes were probed with the corresponding primary antibodies overnight at 4°C (anti-podocin, 1:500, SIGMA P0372; anti-nephrin 1:500, PROGEN GP-N2; anti-WT1, 1:1000, R&D AF5729; anti-Oct3/4, 1:1000 Santa Cruz sc-5279). Horseradish peroxidase-conjugated secondary antibodies, IgGs (1:5000–10.000) were incubated for 1 h at room temperature sequentially. The immunoreactive bands were visualized by a Chemiluminescence kit (ThermoFisher) and images were digitally acquired using LAS-4000 ImageQuant^™^ LAS 4000 Imaging System (GE Healthcare). To assess loading variations, membranes were stripped and re-probed using a rabbit anti-tubulin antibody (1:1000, Santa Cruz sc-80011).

### Albumin uptake assay

Differentiated podocyte cultures were serum-starved overnight. After rinsing the monolayers with PBS, cultures were incubated with 1 mg/ml FITC-conjugated bovine serum albumin for 1 h at 4°C (albumin binding only) and 37°C (binding and endocytosis), respectively, and for 24 h at 37°C. Albumin uptake was determined by fluorescence microscopy, using a ZEISS Axiophot Fluorescence Microscope.

### Doxorubicin toxicity and cell viability assays

In doxorubicin experiments, adult, neonatal, and migraine patient-derived differentiated podocyte-like cells, and undifferentiated and differentiated human immortalized podocytes were incubated for 48 h at concentrations of 10 nM and 1 μM. The resazurin assay was used to assess metabolic activity of the cultures [63]. Resazurin is reduced to resorufin by viable cells. Thus, resazurin reduction is directly proportional to the number of viable cells. Resazurin solution was prepared as a 20-fold stock in PBS from resazurin salt (SIGMA, R7017) and further diluted in culture media. Cultures were incubated for 2 h. The supernatants were transferred into 96 wells for measurement at an excitation wavelength of 540 nm and an emission at 590 nm in a Tecan GENios plus microplate reader.

### Statistical analysis

All experiments were conducted at least three times. Statistical significance was tested using the unpaired Student’s t test. P values <0.05 were considered statistically significant. See legends for more details.

## Supporting information

S1 Fig Immunostaining of podocyte-like cells derived from the iPSC line SFC018 with podocyte markers and F-actin staining. iPSC were differentiated on glass cover slips, fixed and stained for synaptopodin, WT-1, podocin, and F-actin as described in methods. Red and green colours were applied post capture.
(TIF)

S2 Fig Immunostaining of podocyte-like cells derived from the iPSC line SBAD2 with podocyte markers and F-actin staining. iPSC were differentiated on glass cover slips, fixed and stained for synaptopodin, WT-1, podocin, and F-actin as described in methods. Red and green colours were applied post capture.
(TIF)

S3 Fig Time course of induction of podocyte-specific markers during differentiation. Differentiating, SBAD2 and SFC018 iPSC were lysed at days 0 (undifferentiated iPSC, d0) and at days 6 and 13 and processed for Western blot analysis. Representative blots are shown.
(TIF)
