# Supplementary figures and images for: Differentiation of human iPSCs into functional podocytes
Source: PLoS One. 2018 Sep 17;13(9):e0203869. doi: 10.1371/journal.pone.0203869 (PMC6141081; doi:10.1371/journal.pone.0203869)

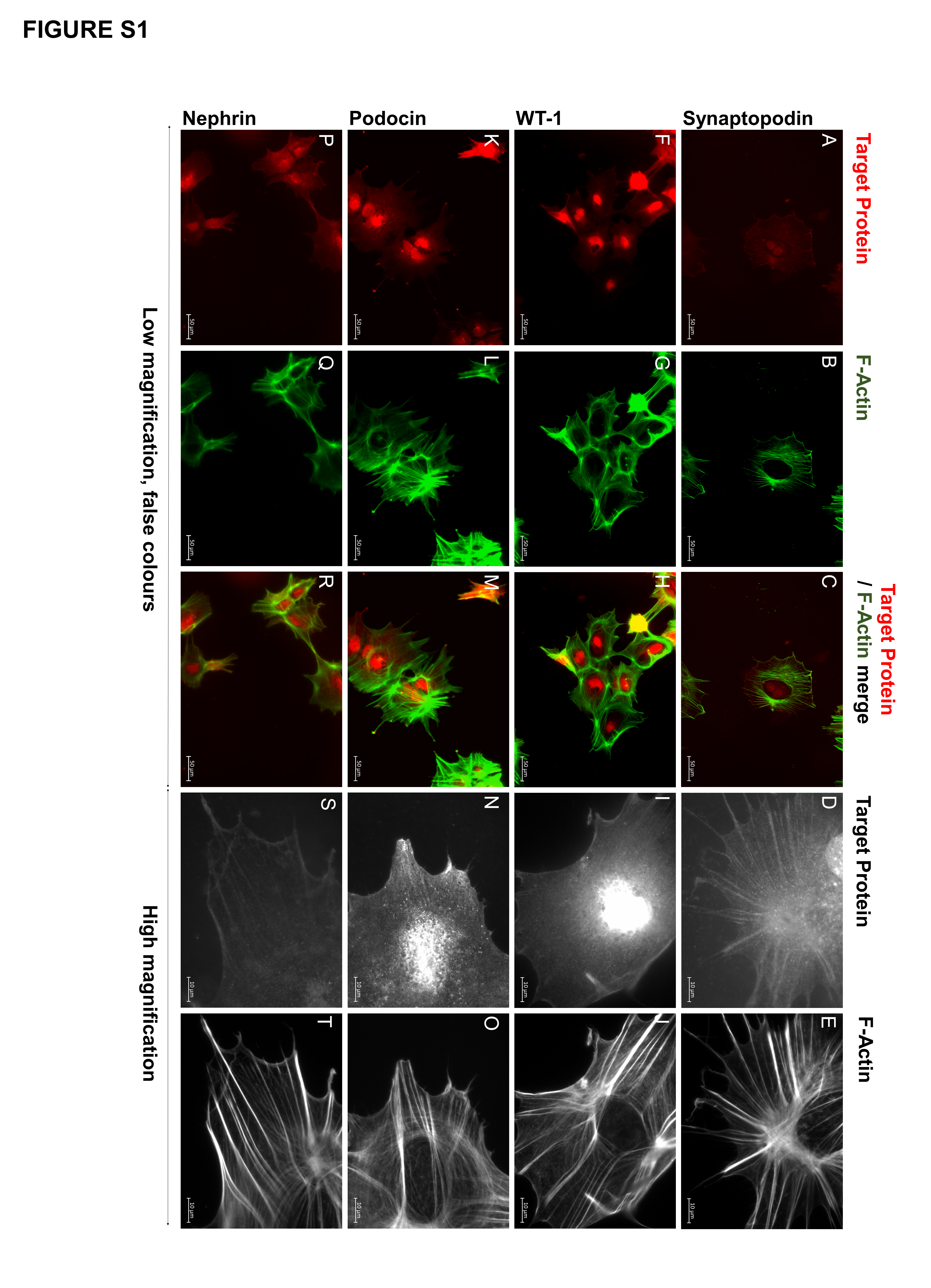

Supplement: S1 Fig — iPSC were differentiated on glass cover slips, fixed and stained for synaptopodin, WT-1, podocin, and F-actin as described in methods. Red and green colours were applied post capture. (TIF) [file pone.0203869.s001.TIF]

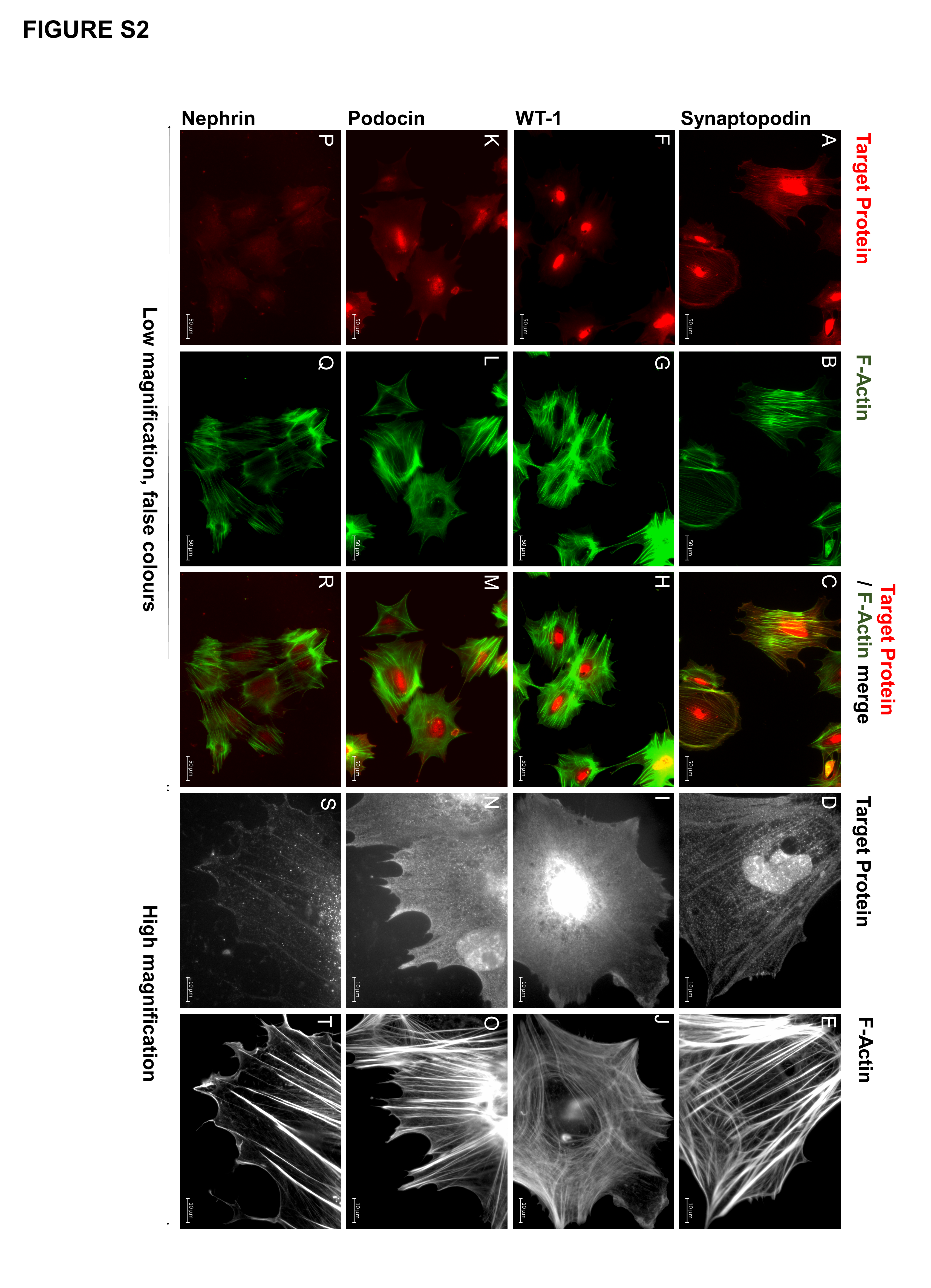

Supplement: S2 Fig — iPSC were differentiated on glass cover slips, fixed and stained for synaptopodin, WT-1, podocin, and F-actin as described in methods. Red and green colours were applied post capture. (TIF) [file pone.0203869.s002.TIF]

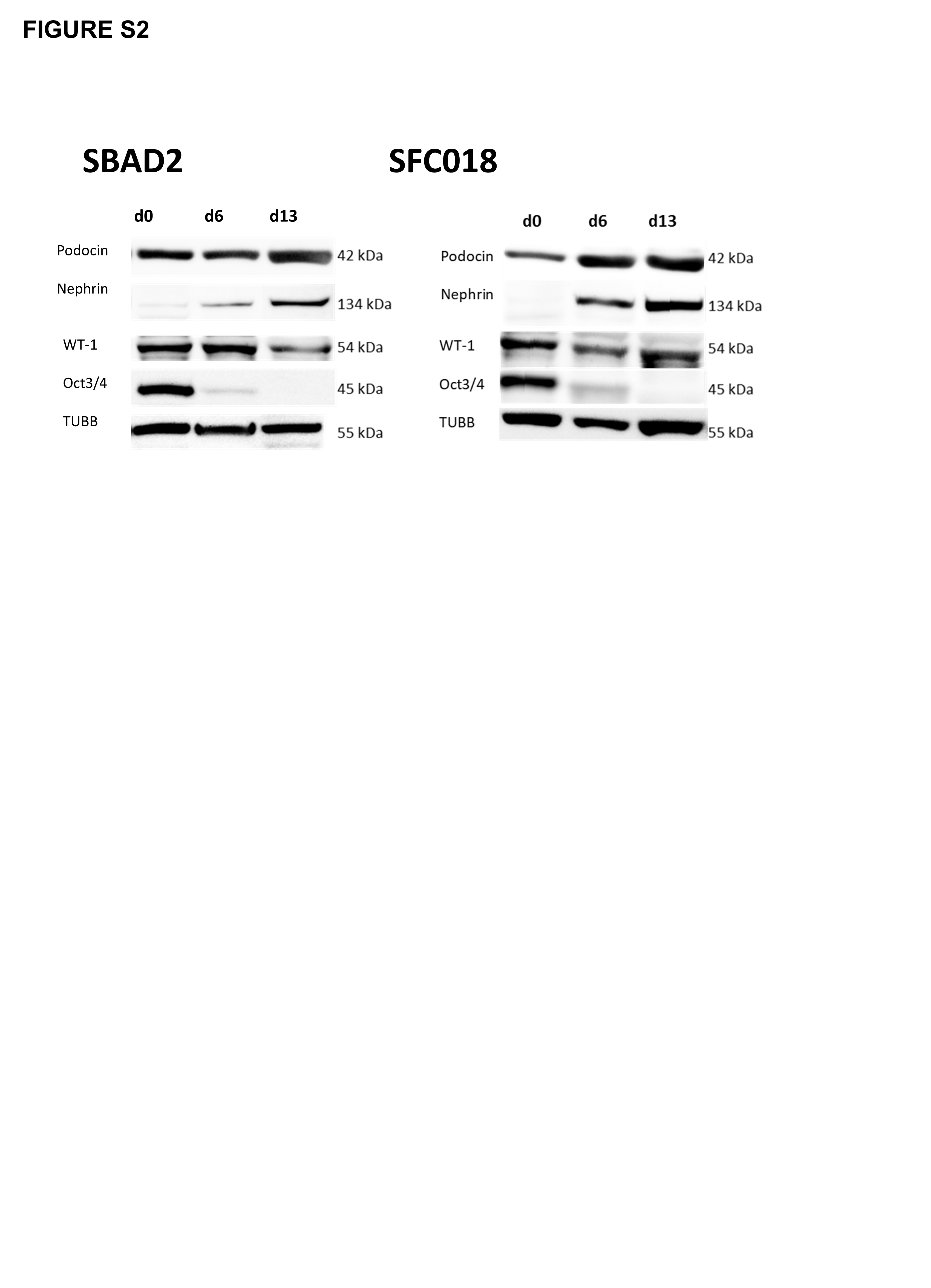

Supplement: S3 Fig — Differentiating, SBAD2 and SFC018 iPSC were lysed at days 0 (undifferentiated iPSC, d0) and at days 6 and 13 and processed for Western blot analysis. Representative blots are shown. (TIF) [file pone.0203869.s003.TIF]
